# Bacteriophages of *Leuconostoc*, *Oenococcus*, and *Weissella*

**DOI:** 10.3389/fmicb.2014.00186

**Published:** 2014-04-28

**Authors:** Witold Kot, Horst Neve, Knut J. Heller, Finn K. Vogensen

**Affiliations:** ^1^Department of Biology, Faculty of Science, University of CopenhagenCopenhagen, Denmark; ^2^Department of Microbiology and Biotechnology, Max Rubner-Institut (Federal Research Institute of Nutrition and Food)Kiel, Germany; ^3^Department of Food Science, Faculty of Science, University of CopenhagenFrederiksberg, Denmark

**Keywords:** bacteriophages, *Leuconostoc*, *Oenococcus*, *Weissella*, morphogenesis, DNA sequence analysis

## Abstract

*Leuconostoc* (*Ln.*), *Weissella*, and *Oenococcus* form a group of related genera of lactic acid bacteria, which once all shared the name *Leuconostoc*. They are associated with plants, fermented vegetable products, raw milk, dairy products, meat, and fish. Most of industrially relevant *Leuconostoc* strains can be classified as either *Ln. mesenteroides* or *Ln. pseudomesenteroides*. They are important flavor producers in dairy fermentations and they initiate nearly all vegetable fermentations. Therefore, bacteriophages attacking *Leuconostoc* strains may negatively influence the production process. Bacteriophages attacking *Leuconostoc* strains were first reported in 1946. Since then, the majority of described *Leuconostoc* phages was isolated from either dairy products or fermented vegetable products. Both lytic and temperate phages of *Leuconostoc* were reported. Most of *Leuconostoc* phages examined using electron microscopy belong to the *Siphoviridae* family and differ in morphological details. Hybridization and comparative genomic studies of *Leuconostoc* phages suggest that they can be divided into several groups, however overall diversity of *Leuconostoc* phages is much lower as compared to, e.g., lactococcal phages. Several fully sequenced genomes of *Leuconostoc* phages have been deposited in public databases. Lytic phages of *Leuconostoc* can be divided into two host species-specific groups with similarly organized genomes that shared very low nucleotide similarity. Phages of dairy *Leuconostoc* have rather limited host-ranges. The receptor binding proteins of two lytic *Ln. pseudomesenteroides* phages have been identified. Molecular tools for detection of dairy *Leuconostoc* phages have been developed. The rather limited data on phages of *Oenococcus* and *Weissella* show that (i) lysogeny seems to be abundant in *Oenococcus* strains, and (ii) several phages infecting *Weissella cibaria* are also able to productively infect strains of other *Weissella* species and even strains of the genus *Lactobacillus*.

## Introduction

### Taxonomy of *Leuconostoc*, *Oenococcus*, and *Weissella*

*Leuconostoc* (*Ln.*), *Weissella* (*W*.), *Oenococcus* (*O*.), and *Fructobacillus* (*F*.) form a group of related genera of lactic acid bacteria. Based on 16S rRNA sequencing, Collins et al. (1993) proposed that *Ln. paramesenteroides* and related species (*Lactobacillus (Lb.) confusus, Lb. halotolerans, Lb. kandleri, Lb. minor, and Lb. viridescens*) should be reclassified in the new genus *Weissella*. Dicks et al. (1995) assigned *Ln. oenos* to the new genus *Oenococcus*. Endo and Okada (2008) proposed to allocate several *Leuconostoc* species to the new genus *Fructobacillus*. Schleifer (2009), on the basis of 16S rRNA sequences, transferred the three genera *Leuconostoc* (including those species synonymous with *Fructobacillus*), *Weissella*, and *Oenococcus* into the newly formed family *Leuconostocaceae*. Members of the family show highest similarity to the genus *Lactobacillus*: they all are Gram-positive, catalase-negative, facultative anaerobes, and are characterized by heterofermentative lactic acid fermentation. While all members of the genera *Leuconostoc* and *Oenococcus* exhibit ovoid-shaped morphology, members of the genus *Fructobacillus* are rod-shaped. Species within the genus *Weissella* show two different (i.e., rod-shaped and ovoid-shaped) morphotypes.

### Species in the family *Leuconostocaceae*

According to information presented on the web-site of the “List of prokaryotic names with standing in nomenclature” (http://www.bacterio.net) the genus *Leuconostoc* is represented by 23 species and 4 subspecies for *Ln. mesenteroides*. However, several of the species names are synonyms within the genus *Leuconostoc* (like, e.g., *Ln. argentinum* and *Ln. lactis*, or *Ln. mesenteroides* subsp. *cremoris* and *Ln. cremoris*) or within different genera (like, e.g., *Ln. pseudoficulneus* and *F. pseudoficulneus*, *Ln. paramesenteroides* and *W. paramesenteroides*, *Ln. oenos* and *O. oeni*). Besides being recognized as meat spoilage organisms, *Leuconostoc* species have been described to be involved in several fermentation processes (Björkroth and Holzapfel, 2006). *Ln. mesenteroides* subsp. *mesenteroides*, *Ln*. *mesenteroides* subsp. *cremoris*, *Ln. lactis*, and *Ln. pseudomesenteroides* are regular constituents of aroma-producing starter-cultures applied in dairy fermentations (Farrow et al., 1989). In addition, *Ln. mesenteroides* subsp. *mesenteroides* is an important component of vegetable fermentations: it is involved in fermentation of coffee beans and (together with *Ln. fallax*) in sauerkraut fermentation. *Ln. mesenteroides*, * Ln. citreum*, *Ln. gelidum* and *Ln. kimchii* are dominant species in early kimchi fermentation, *and Ln. mesenteroides* subsp. *dextranicum* plays a key role in sourdough fermentations (Schleifer, 2009).

The genus *Weissella* comprises 18 species. As already mentioned for the genus *Leuconostoc*, some of the species names are synonyms within the genus (like, e.g., *W. cibaria* and *W. kimchii*) or within different genera (like the above mentioned *Lactobacillus* species proposed to be reclassified as *Weissella*). The species *W. cibaria*, *W. confuse*, and *W. koreensis* have been described to be associated with vegetable fermentations (Schleifer, 2009). Recently, *W. fabalis* and *W. paramesenteroides* have been detected in cocoa bean fermentation (Snauwaert et al., 2013) and in traditional Caciocavallo cheese (Settanni et al., 2012), respectively.

The genus *Oenococcus* comprises just two species: *O. oeni* originally described as *Ln. oenos*, and *O. kitaharae* isolated from composting residues of schochu distillation (Endo and Okada, 2006). *O. oeni* plays an important role in wine fermentation, where it decarboxylates malic acid to lactic acid (Schleifer, 2009).

The genus *Fructobacillus* is represented by five species, all of which except *F. tropaeoli* are synonyms of *Leuconostoc* species (Endo et al., 2011). Species of this genus have been described to be involved in spontaneous cocoa bean fermentations (Snauwaert et al., 2013). So far, no bacteriophages have been described for this genus. Therefore, the fructobacilli will not be further addressed in this review.

### History of bacteriophages in the family *Leuconostocaceae*

The first description of bacteriophages affecting *Leuconostoc* was published in 1946 (Mosimann and Ritter, 1946). In this publication already, the negative impact on butter aroma of bacteriophages infecting *Leuconostoc* strains was shown. Just 1 year later, Leiva-Quiros and McCleskey (1947) isolated phages infecting *Ln. mesenteroides* for phage-typing purposes. From late 1970's to beginning of 2000's only dairy *Leuconostoc* phages have been reported on with the exception of one report also included phages from coffee fermentations (Table 1) (Boizet et al., 1992). From 2002 to 2012 a number of reports on *Leuconostoc* phages from sauerkraut fermentations have been published, and since 2010–2012 genomes of *Ln. mesenteroides* and *Ln. pseudomesenteroides* phages have been published (Table 1). A thorough classification of dairy *Leuconostoc* phages has been presented recently (Ali et al., 2013).

**Table 1:** **Table summarizing reports on phages infecting genus *Leuconostoc***.

***Leuconostoc* host species**	**Origin**	**Life style**	**Analysis**	**References**
*Ln. citrovorum* (*Ln*. *mesenteroides* subsp. *cremoris*)	Dairy	Lytic	Flavor defects	Mosimann and Ritter, 1946
*Ln. mesenteroides*	Dairy	Lytic	TEM (2 phages)	Sozzi et al., 1978
*Ln. mesenteroides* subsp. *cremoris*, subsp *dextranicum*, and subsp. *mesenteroides*	Dairy	Lytic (4 phages)	Host range	Shin and Sato, 1979
*Ln. mesenteroides* subsp. *dextranicum* and subsp. *mesenteroides*	Dairy	Temperate	TEM (9 phages)	Shin and Sato, 1979
*Ln. mesenteroides* subsp. *cremoris*	Dairy	Lytic (phage Lc-4)	1-step growth	Shin, 1983
*Ln. mesenteroides* subsp. *cremoris*	Dairy	Lytic (4 phages)	Host range, TEM	Saxelin et al., 1986
*Ln. mesenteroides* subsp. *cremoris*	Dairy	Lytic (phage PWL-2)	TEM, structural proteins, REN analysis	Neve et al., 1988
*Ln. mesenteroides* subsp. *cremoris* and subsp. *Ln. lactis*	Dairy	Lytic (4 phages)	Host range	Johansen and Kibenich, 1992
*Ln. mesenteroides*	Dairy, coffee	Lytic (19 phages)	6 DNA homology groups, structural proteins, TEM, REN analysis, genome sizes	Boizet et al., 1992
*Leuconostoc* sp.	Dairy	Lytic (4 phages)	1 DNA homology group, structural proteins, TEM, REN analysis	Davey et al., 1995
*Ln. mesenteroides* and *Leuconostoc sp.*	Sauerkraut	Lytic (8 phages)	TEM, Host range	Yoon et al., 2002
*Ln. fallax*	Sauerkraut	Lytic (6 phages)	TEM, REN analysis, Host range, structural proteins	Barrangou et al., 2002
*Ln. mesenteroides, Ln. citreum, Ln. pseudomesenteroides, Ln. fallax*	Sauerkraut	Lytic (29 phages)	Host range (all), TEM, REN analysis, structural proteins (6 phages)	Lu et al., 2003
*Ln. mesenteroides*	Sauerkraut	Lytic (Φ1-A4)	TEM, structural proteins, genome sequence (29.5 kb)	Lu et al., 2010
*Ln. pseudomesenteroides*	KC04 strain	Temperate (ΦMH1)	TEM, genome sequence (38.7 kb)	Jang et al., 2010
*Ln. mesenteroides; Ln. pseudomesenteroides*	Dairy	Lytic (77 phages)	Host range, thermal stability and inactivation kinetics, TEM	Atamer et al., 2011
*Ln. mesenteroides (pseudomesenteroides)*	Dairy	Lytic (phage Lmd1)	TEM, genome sequence (26.2 kb)	Kleppen et al., 2012a
*Ln. mesenteroides* and *pseudomesenteroides*	Dairy	Lytic (83 phages)	TEM, 2 DNA homology groups, host range, PCR detection	Ali et al., 2013
*Ln. pseudomesenteroides*	Dairy	Lytic (2 phages)	TEM, receptor binding proteins	Kot et al., 2013
*Ln. mesenteroides* and *Ln. pseudomesenteroides*	Dairy	Lytic (9 phages)	TEM, genome sequence, structural proteins, host range	Kot et al., 2014
*Ln. mesenteroides* subsp. *mesenteroides*	Dairy	Lytic (9 phages)	TEM, stability and inactivation kinetics, REN analysis, host range	Pujato et al., 2014

Type of analysis presented in the paper is listed in the column “analysis.” TEM, Transmission electron microscopy, REN, analysis of restriction endonuclease fragments.

Lu et al. (2003) reported on bacteriophages infecting *Weissella* sp. Later, several studies described *Podoviridae*-phages infecting *W. cibaria* (Pringsulaka et al., 2011; Kleppen et al., 2012b).

Sozzi et al. (1976) were the first to describe phage infecting lactic acid bacteria in wine, which were later identified as *O. oeni* (Sozzi et al., 1982; Dicks et al., 1995). Lysogeny appears to be rather frequent in *O. oeni*, with 45–60% of *O. oeni* strains reported to be lysogenic (Arendt et al., 1991; Poblet-Icart et al., 1998). Pan-genome comparisons have confirmed these results and have demonstrated that apparently six different bacterial tRNA genes are involved as targets for prophage DNA integration of temperate bacteriophages in different strains of *O. oeni* (Borneman et al., 2012).

### Fermentations affected by phages infecting species of *Leuconostocaceae*

Dairy fermentations are the most frequently described fermentations affected by bacteriophages (Samson and Moineau, 2013). This may be due to two major reasons: (i) milk is a liquid substrate in which phage are easily distributed, and (ii) most dairy fermentations involve application of starter culture mixtures, thus variations in acidification performance become readily evident. Presence of phages infecting dairy *Leuconostoc* strains has only been described occasionally (Sozzi et al., 1978; Boizet et al., 1992; Davey et al., 1995). However, the publications never acquired attention similar to those describing phages causing disturbances of acidification. This is probably due to the fact that during fermentation acidification failures are much easier and much earlier detectable than aroma defects. As a consequence, while the negative impact of phage on starter strains and acidification is well documented, the impact of phage on starter strains and aroma development is much less well known and is only beginning to be investigated systematically (Samtlebe et al., 2012). In recent years Swedish and Danish dairies have reported problems related to lack of diacetyl and CO_2_ in fermented milks (similar to report by Mosimann and Ritter, 1946) that could be correlated to phages attacking *Leuconostoc* strains (Anon. meeting reports, Vogensen, not published). Similarly, in several cases phage attacks on *Leuconostoc* strains in blue-mold cheeses have been correlated with lack of mold growth probably due to less openness in the cheese structure (Kot et al., 2014; Pujato et al., 2014).

**Figure 1:**
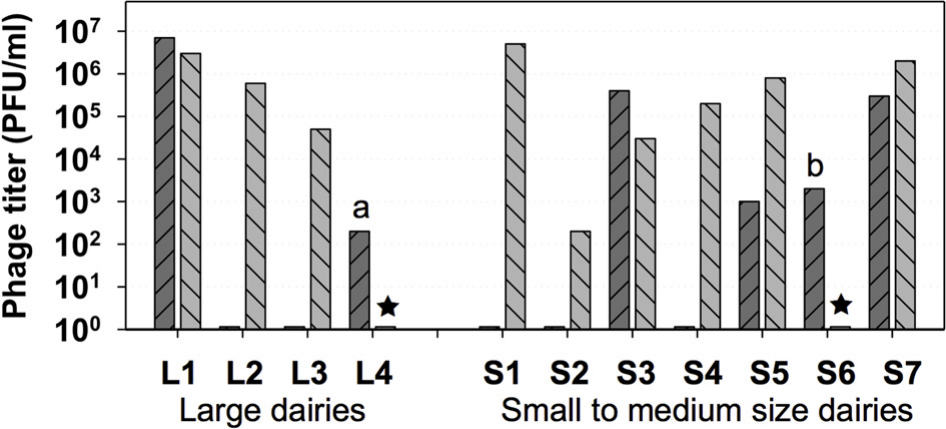
**Highest *Leuconostoc* phage titers in whey (**
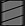
**) and in brine (**
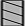
**) samples obtained from 4 large and from 7 small to medium size dairies**. ★, no brine samples (dairies L4 and S6); a, butter milk sample; b, butter cream sample. (Modified from Atamer et al., 2011).

Highest *Leuconostoc* phage titers in dairy products or in whey samples can vary significantly within a range from approximately 10^2^ to 10^7^ plaque-forming units (PFUs) per gram or per ml (Atamer et al., 2011) (Figure 1). The maximum numbers of PFUs for *Leuconostoc* phages in dairy samples are approximately 2 log units lower than maximum lactococcal phage numbers (approximately 10^9^ PFU/ml). The lower *Leuconostoc* phage numbers are therefore probably reflecting the use of *Leuconostoc* as a minor starter component (1–10%) in undefined complex cultures consisting mainly of lactococcal strains. While the homo-lactic lactococci mainly contribute to acidification, the heterolactic and only weakly acidifying leuconostocs contribute to aroma by production of acetate, acetoin, and diacetyl (Farkye and Vedamuthu, 2002).

The *Leuconostoc* lytic phages involved in dairy fermentations have generally been shown to be members of the *Siphoviridae* group of phages (Davey et al., 1995; Kleppen et al., 2012a; Ali et al., 2013; Kot et al., 2014; Pujato et al., 2014). However, for *Leuconostoc* phages isolated from sauerkraut fermentations also *Myoviridae* phages are seen (Barrangou et al., 2002; Yoon et al., 2002; Lu et al., 2003). Other than in dairy fermentations, in sauerkraut fermentations phages may even play an important role by affecting the development of different lactic acid bacteria species over fermentation time (Lu et al., 2003). However, when defined starter strains are supposed to be applied, phage infection may negatively affect quality parameters of the final product (Mudgal et al., 2006). Applying metagenomic analysis to kimchi, a traditional Korean fermented cabbage, evidence for presence of phage infecting *Leuconostoc* was obtained (Jung et al., 2011). So far, the only phage/host pair characterized for kimchi is a *Podoviridae* phage infecting *Weissella cibaria* (Kleppen et al., 2012b). A similar pair, *Podoviridae* phage and W*. cibaria* host, has been described for Nham, a Thai fermented pork sausage (Pringsulaka et al., 2011). Recently, several phages infecting *W. cibaria* and *W. paramesenteroides* were isolated from commercial cucumber fermentations and one phage for each host was shown to belong to the *Siphoviridae* family of phages (Lu et al., 2012). The only fermentation known to be affected by phages infecting *O. oeni* is wine fermentation, due to the exclusive involvement of these host bacteria in this type of fermentation (Schleifer, 2009). The phages have been shown to belong to the *Siphoviridae* family of phages (Poblet-Icart et al., 1998).

## *Leuconostoc* phages

Phages attacking *Leuconostoc* are best documented among all phages of the *Leuconostocaceae* family. The majority of reports on *Leuconostoc* phages are connected to problems in dairy fermentations, however few of the reports are dealing with *Leuconostoc* phages in vegetable or in coffee fermentations (Table 1). However, *Leuconostoc gelidum* is a known meat spoilage organism (Sakala et al., 2002). Interestingly, it was proposed to use *Ln. gelidum* phages to prevent bacterial spoilage of the meat products, pointing toward a different angle of phage-host interactions, i.e., phage bioprotection in fermented foods (Greer et al., 2007).

### Morphology of *Leuconostoc* phages

**Figure 2:**
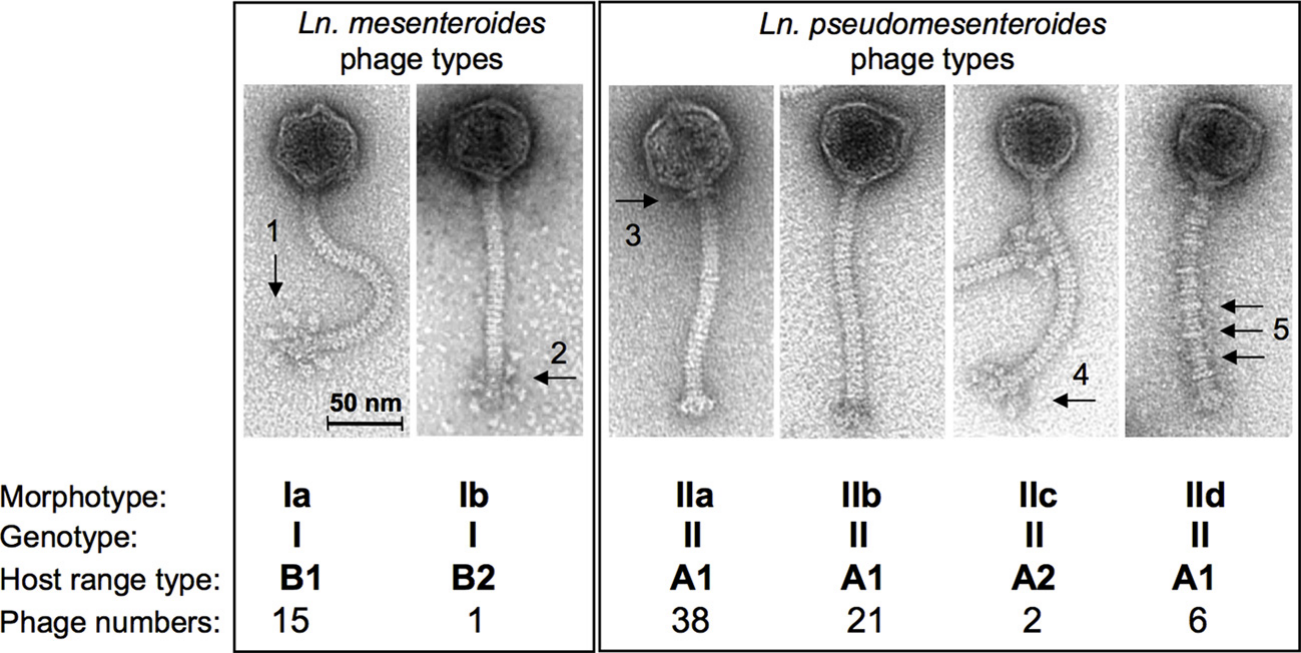
**Overview on the current taxonomy of dairy *Leuconostoc* phages based on transmission electron microscopy, genotyping, and host range profiles**. Arrows indicate structural details as follows: globular baseplate appendices (1), non-globular (Y-shaped) baseplate appendages (2), collar or neck passage structure (3), “fluffy” baseplate appendices (4), tail striations (5). (modified from Ali et al., 2013).

Recently, the morphotypes of dairy *Ln. pseudomesenteroides* and of *Ln. mesenteroides* phages from the dairy environment have been studied extensively with a set of 83 phage isolates (Ali et al. (2013). Although the phages were isolated from various sources (11 dairies, 3 phage collections), a low degree of variation was documented for their morphotypes. All phages were small isometric-headed *Siphoviridae* phages with non-contractile 140-nm long tails, however, according to their baseplate structure, these phages were differentiated into 6 different subgroups with six globular baseplate appendices or with peculiar Y-shaped baseplate structures (*Ln. mesenteroides* phages of morphotypes Ia or Ib), with plain baseplates but with or without characteristic collar structures or with uncommon tail striations (*Ln. pseudomesenteroides* phages of morphotypes IIa, b and d), or with undefined “fluffy” baseplate appendices (morphotype IIc *Ln. pseudomesenteroides* phages) (Figure 2). Dairy *Leuconostoc* phages of morphotypes Ia and IIb had been reported occasionally, i.e., Ia type phages: (Neve et al., 1988); IIb type phages: (Saxelin et al., 1986; Davey et al., 1995; Kleppen et al., 2012a). *Siphoviridae* phages of *Leuconostoc* with longer phage tails have also been described previously, indicating a broader biodiversity (Saxelin et al., 1986) within *Leuconostoc* phage populations. This correlates well with the establishment of 6 DNA homology groups for *Ln. mesenteroides* phages (Boizet et al., 1992). *Leuconostoc* phages isolated from sauerkraut fermentations did also reveal different morphotypes *Siphoviridae* phages with different tail lengths and *Myoviridae* phages (Barrangou et al., 2002; Lu et al., 2003, 2012). Temperate *Siphoviridae* phages from lysogenic *Ln. mesenteroides* and *Ln. pseudomesenteroides* strains with different tail lengths have been shown by Shin and Sato (1979), Lu et al. (2012), and Jang et al. (2010).

### Genetics of *Leuconostoc* phages

Currently, there are 12 full genomes of phages infecting *Leuconostoc* sp. present in publically available databases. All phages have dsDNA genomes with sizes from 25.7 to 38.7 kb (Table 2). Genomic G + C content varies from 36.1% for phage Φ-A4 to 38.7% for phage ΦMH1. All described lytic phages of *Leuconostoc* exhibit high similarity in regard to genome organization. Five modules can be distinguished in the genomes specifying replication, packaging, morphogenesis, host cell lysis, and regulation and modification. Moreover, high similarity of putative proteins encoded in the genomes of lytic *Leuconostoc* phages suggests that they originated from a common ancestor (Kot et al., 2014). Ali et al. (2013) reported two groups of hybridization patterns among lytic *Leuconostoc* phages of dairy origin; one for *Ln. mesenteroides* phages and one for *Ln. pseudomesenteroides* phages, although all phage members of the two groups share a short, cross-hybridizing genome region. The cross-hybridizing region codes for tail proteins, e.g., major tail protein (*mtp*) and tape measure protein (*tmp*), however higher similarities were found within *mtp* gene. This conserved region was used as target for developing a universal PCR-based detection system for lytic phages of *Ln. mesenteroides* and *Ln. pseudomesenteroides* (Ali et al., 2013). The PCR assay resulted in 322-bp long fragments and was validated with all reported 83 lytic phages of *Leuconostoc* (Ali et al., 2013). The recent sequencing data confirms that the selected region is indeed the only region that can be used for PCR-based detection for both phage species (Kot et al., 2014).

**Table 2:** **Bacteriophages of *Leuconostoc* and *Weissella* with complete genome sequences deposited in public databases**.

**Genus**	**Name**	**Host^a^**	**Accession nr**	**Information**	**Genome size (kb)**	**References**
*Leuconostoc*	Φ1-A4	*Ln. mesenteroides* 1-A4	GQ451696	Lytic, *cos*-type	29.5	Lu et al., 2010
*Leuconostoc*	ΦLmd1	*Ln. mesenteroides* ssp. *dextranicum* A1	NC_018273	Lytic, *cos*-type	26.2	Kleppen et al., 2012a
*Leuconostoc*	ΦLN25	*Ln. mesenteroides* LN25	KC013026	Lytic, *cos*-type	28.4	Kot et al., 2014
*Leuconostoc*	ΦLN34	*Ln. mesenteroides* LN05	KC013027	Lytic, *cos*-type	28.0	Kot et al., 2014
*Leuconostoc*	ΦLNTR2	*Ln. mesenteroides* LN05	KC013028	Lytic, *cos*-type	28.3	Kot et al., 2014
*Leuconostoc*	ΦLNTR3	*Ln. mesenteroides* LN05	KC013029	Lytic, *cos*-type	28.0	Kot et al., 2014
*Leuconostoc*	P793	*Ln. pseudomesenteroides* BM2	NC_020880	Lytic, *cos*-type	26.8	Kot et al., 2013
*Leuconostoc*	ΦLN04	*Ln. pseudomesenteroides* LN02	NC_020870	Lytic, *cos*-type	25.9	Kot et al., 2013
*Leuconostoc*	ΦLN03	*Ln. pseudomesenteroides* LN02	KC013022	Lytic, *cos*-type	26.8	Kot et al., 2014
*Leuconostoc*	ΦLN12	*Ln. pseudomesenteroides* LN02	KC013025	Lytic, *cos*-type	28.2	Kot et al., 2014
*Leuconostoc*	ΦLN6B	*Ln. pseudomesenteroides* LN02	KC013024	Lytic, *cos*-type	25.7	Kot et al., 2014
*Leuconostoc*	ΦMH1	NA^a^	HM596271	Induced from *Ln. pseudomesenteroides* KC04	38.7	Jang et al., 2010
*Weissella*	ΦYS61	*Weissella cibaria* YS61	NC_018270	Lytic, protein dependent DNA packaging	33.6	Kleppen et al., 2012b

Genomes of lytic phages of *Leuconostoc* contain from 38 to 50 predicted genes. Some of us were involved in biological characterization of one of the genes present in *Ln. pseudomesenteroides* phages, namely the receptor binding protein (RBP) (Kot et al., 2013). Construction of chimeric phages resulted in the transition in host range allowing the identification of the receptor binding protein genes to be ORF21_P793_ and ORF23_Φ LN04_, respectively. Until now, the host-encoded receptor for *Leuconostoc* phages remains unknown.

**Figure 3:**
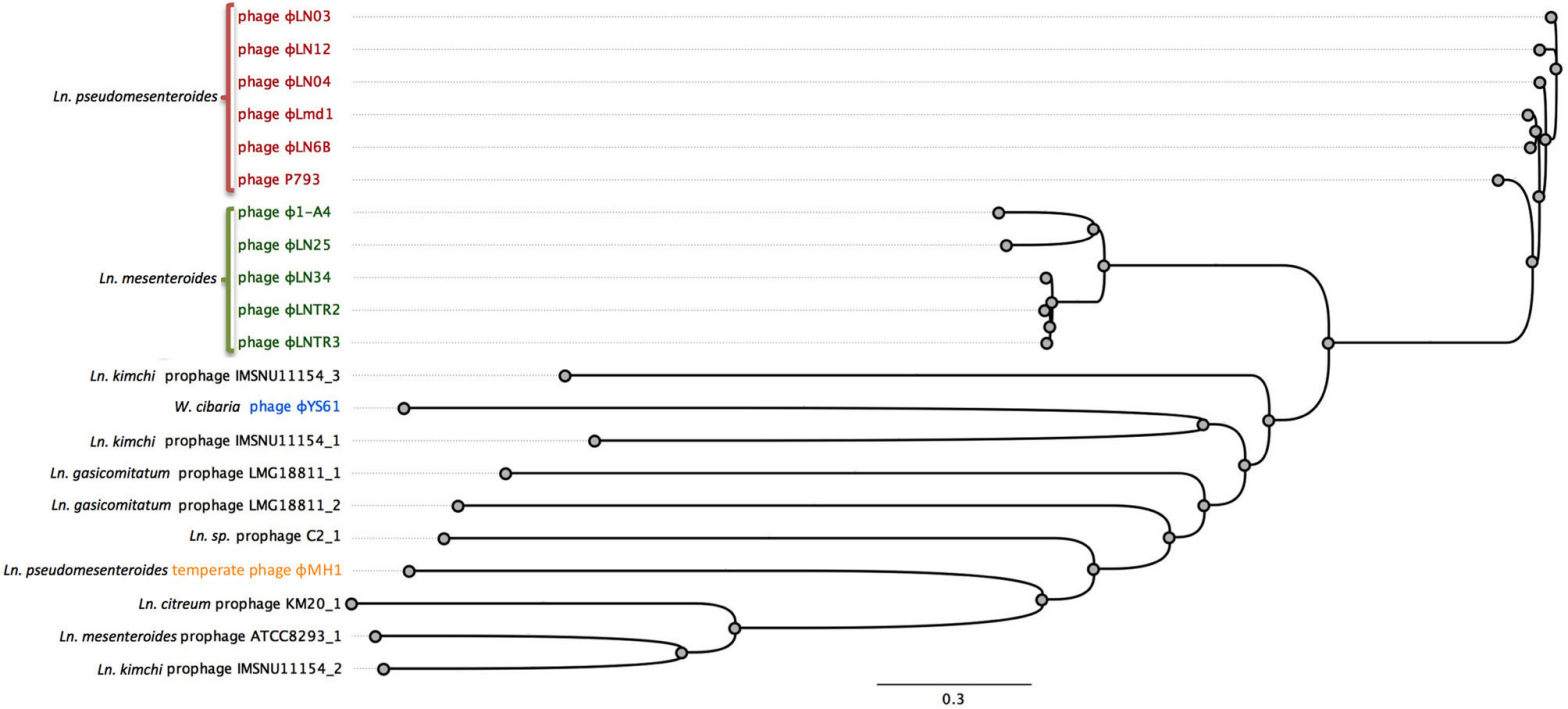
**Comparison of the complete translatome of fully sequenced phages and predicted prophages from complete genomes of *Leuconostoc*, *Weissella*, and *Oenococcus***. Genomes were prepared as described before (Mahony et al., 2013). The concatemeric sequences were aligned using Geneious 7.1.2 software using BLOSUM62 cost matrix. The phylogenetic tree was constructed and visualized with bootstrapped neighbor-joining method with 500 replicates in Geneious 7.1.2 software.

Currently, there is only one complete genome sequence of a temperate phage attacking *Leuconostoc* deposited in public databases. The phage is designated ΦMH1 and it was obtained from a UV-induced lysate of *Ln. pseudomesenteroides* strain KC04 (Jang et al., 2010). No host for ΦMH1 phage was reported. ΦMH1 has a dsDNA genome with a length of 38.7 kb with 65 putative ORFs identified. ΦMH1 did not show significant similarities with other described phages of *Leuconostoc* (Jang et al., 2010). Besides of ΦMH1 phages, several predicted prophages can be identified in the sequenced genomes of *Leuconostoc* (Table 3). Analysis of complete translatome of fully sequenced phages and prophages shows that diversity of prophage elements is higher than within sequenced two groups of lytic phages (Figure 3).

**Table 3:** **Predicted prophage sequences found in fully assembled chromosomes of *Leuconostoc, Oenococcus*, and *Weissella* available in GenBank**.

**Species**	**Strain**	**Accession nr**	**Number of predicted prophages**	**Size of predicted prophages (kb)**
*Leuconostoc mesenteroides*	ATCC 8293	NC_008531	1	41.9
*Leuconostoc mesenteroides*	J18	NC_016805	0	
*Leuconostoc citreum*	KM20	NC_010471	1	50.5
*Leuconostoc gelidum*	JB7	NC_018631	0	
*Leuconostoc gasicomitatum*	LMG 18811	NC_014319	2	11.5, 45.1
*Leuconostoc* sp.	C2	NC_015734	1	37.5
*Leuconostoc carnosum*	JB16	NC_018673	0	
*Leuconostoc kimchi*	IMSNU 11154	NC_014136	3	13.1, 36.8, 65
*Oenococcus oeni*	PSU-1	NC_008528	0	
*Oenococcus kitaharae*	DSM 17330	NZ_CM001398	0	
*Weissella koreensis*	KACC 15510	NC_015759	0	

Prediction was done using PHAST (Zhou et al., 2011) and PhiSpy (Aziz et al., 2008; Akhter et al., 2012) and manually verified afterwards.

## *Oenococcus* phages

Phages attacking *O. oeni* were reported already in the late 1960's and beginning of 1970's (Sozzi et al., 1976), and 3 phage morphotypes were described for phages isolated from wine. They all had isometric heads and 3 distinct non-contractile tail lengths, i.e., belonging to *Siphoviridae*. Later, lytic phages from four Australian wine areas attacking approximately 40% of *O. oeni* isolates from the same regions were isolated (Davis et al., 1985). These also were belonging to *Siphoviridae* with isometric heads and long non-contractile tails (approximately 300 nm). Subsequently, phage P58I was isolated from a phage carrying culture of *O. oeni* 58N (Arendt et al., 1990). This phage was able to plaque on strain 58N as well as on 58PF, which was a phage-free derivative of 58N strain. A similar phage P58II isolated after mitomycin C induction of the 58N strain was not able to plaque on any of the two strains. Surprisingly no DNA homology was detected between the two phages genomes and the chromosome of *O. oeni* 58N.

Huang et al. isolated a temperate phage Φ1002 that was able to grow lytically on approximately 46% of all *O. oeni* isolates from Australian wine (Huang et al., 1996). The phage belonged to the *Siphoviridae* family with a 52 nm isometric head and a 210 nm non-contractile tail. A set of 17 prophages were induced from *O. oeni* isolated from Portuguese wines (Santos et al., 1998). They all had a similar morphology with isometric heads of approximately 40–50 nm and non-contractile tails of approximately 220–240 nm. The *cos*-type phages were divided into 6 groups based on restriction enzyme digestion profiles. These could further be divided into 2 main groups α and β based on restriction maps (Santos et al., 1998). Cross-hybridization between the α and β group was located in the central part of the genomes and included the phage attachment site (*attP*). This part was later sequenced (Parreira et al., 1999) and revealed the presence of lysin and holin genes.

The lysin (Lys44) from *O. oeni* phage fOg44 was described in greater detail. Interestingly, secretion of the lysin seems to occur with the aid of a signal peptide and independent of the holin, also encoded in the phage genome. A potential role of the holin as a triggering factor for lytic activity is discussed (São-José et al., 2000).

Screening of 167 isolates of *O. oeni* for lysogeny by mitomycin C resulted in the identification of approximately 45% of lysogenic strains and for some of these propagating hosts were identified as indicator strains (Poblet-Icart et al., 1998).

Until now, there is no complete genome sequence of a phage attacking *O. oeni*, however a number of partial sequences derived from phages of *O. oeni* have been deposited in public databases. Borneman et al. (2012) reported several prophage sequences in the *O. oeni* pan genome. Prophage-like sequences were integrated into six different tRNA genes, with some of these sequences representing presumably functional phages (Borneman et al., 2012). Recently, Doria et al. (2013) communicated a PCR-based method for detection and identification of lysogenic strains of *O. oeni*. The assay allowed detection of a target sequence within the prophage lysin gene in 25 out of 37 isolates tested. Furthermore, the majority of the lysogenic isolates could be prophage induced (Doria et al., 2013). Shortly after, Jaomanjaka et al. (2013) analyzed oenococcal prophages based on integrase gene polymorphism and classified them into four groups (A–D). Remarkably, in the two fully assembled chromosomes of *Oenococcus* sp. no prophage sequences were detected using PHAST and PhiSpy program (Aziz et al., 2008; Zhou et al., 2011; Akhter et al., 2012) (Table 3). Absence of prophage-like sequences in the *O. oeni* PSU-1 strain had been reported before by Mills et al. (2005).

## *Weissella* phages

Pringsulaka et al. (2011) isolated phage Φ22 attacking *Weissella cibaria* N22 from a Thai fermented pork sausage Nham. This phage belonged to the *Podoviridae* family with morphotype C2 with a prolate head of approximately 92 × 50 nm and a non-contractile tail of 37 nm. Phage Φ22 had a narrow host-range attacking only one of 40 *W. cibaria* strains.

Lu et al. (2012) also isolated phages attacking *W. cibaria* from the initial phase of cucumber fermentation. Interestingly, the host range of some of these phages crossed the species barrier and in some cases also the genus barrier. Phage Φ3.8.18 belonging to the *Myoviridae* family attacked two isolates of *W. cibaria*, one isolate of *Lb. plantarum* and one isolate of *Lb. brevis*. Phage Φ3.8.18 had an isometric head of approximately 80 nm and an approximately 200 nm tail with indication of a baseplate structure. Another *Myoviridae* phage Φ7.2.50 attacked same two isolates of *W. cibaria* and 24 isolates of *Lb. brevis*. Also two *Siphoviridae* phages crossed the species/genus barrier. Phage Φ3.8.43 attacked, beside four *W. cibaria* isolates, one isolate of *Lb. plantarum*, and one isolate of *Lb. brevis* (both of which were also attacked by Φ3.8.18). Phage Φ3.8.43 had an isometric head of approximately 50–60 nm and an approximately 250 nm long non-contractile tail. Besides two isolates of *W. cibaria*, phage Φ3.8.48 also attacked one isolate of *W. paramesenteroides*. Kleppen et al. (2012b) determined the genome sequence of ΦYS61 attacking *W. cibaria* (Table 2). This phage isolated from 1-week old kimchi fermentation belonged to the *Podoviridae* family of morphotype C2 (Ackermann, 1998) with a prolate head of 85 × 36 nm and a short non-contractile tail.

Phage ΦYS61 is infecting *W. cibaria* (Kleppen et al., 2012b) and has a 33.6 kb dsDNA genome, which is similar to the estimated genome size of another podovirus of *W. cibaria* isolated recently from a Thai sausage (Pringsulaka et al., 2011). The genome of the ΦYS61 phage codes for 48 putative ORFs. It is very likely that ΦYS61 utilizes a protein-dependent DNA replication mechanism similarly to Φ29 phage from *Bacillus subtilis* (Kleppen et al., 2012b). Very few putative genes of ΦYS61 show significant similarities to the sequences present in public databases. No prophages were detected in *W. koreensis* KACC 15510 strain (Table 3).

## Conclusion

Phages of *Leuconostoc*, *Oenococcus*, and *Weissella* are present in many types of food-related fermentations, where they are responsible for various defects in production. The majority of described phages were isolated from dairy samples, where they attack *Leuconostoc* starter strains and subsequently contribute to aroma- and CO_2_-production defects. Another large reservoir of *Leuconostoc* and *Weissella* phages are various vegetable fermentations, most importantly kimchi and sauerkraut fermentations. All phages of *Oenococcus* described so far are solely reported in connection to wine production, where they can disturb the malolactic fermentation.

All phages of *Leuconostoc*, *Oenococcus*, and *Weissella* belong to *Caudovirales* order with members of the *Siphoviridae*, *Podoviridae*, and *Myoviridae* families. Thirteen complete genomes of phages infecting *Leuconostoc* and *Weissella* have been reported. Among them, lytic phages of *Leuconostoc* belonging to *Siphoviridae* exhibit high similarities in overall composition, regardless on the environment they were isolated from. PCR-based assays for detecting lytic *Leuconostoc* and *Oenococcus* phages have been established so far, however further detailed knowledge of the genetic diversity of *Leuconostoc*, *Oenococcus*, and *Weissella* phages, e.g., *Myoviridae* phages from sauerkraut fermentations as well as temperate phages is needed in order to provide better taxonomy, control, and detection strategies for these groups of phages.

### Conflict of interest statement

The authors declare that the research was conducted in the absence of any commercial or financial relationships that could be construed as a potential conflict of interest.
